# Comparison of the effects of hydrogel and normal saline as carriers of MSC on fracture healing in a rat long bone fracture model

**DOI:** 10.1186/s13018-025-06029-y

**Published:** 2025-07-08

**Authors:** Kang-Il Kim, Ki-Hyeok Ku, Hyun-Mi Cho, Hyeon-Gyu Han, Myung-Seo Kim

**Affiliations:** 1 Department of Orthopedic Surgery, Kyung Hee University College of Medicine, Kyung Hee University Hospital at Gangdong, Seoul, 05278 Republic of Korea; 2 Department of Core Research Laboratory, Clinical Research Institute, Kyung Hee University Hospital at Gangdong, Seoul, 05278 South Korea

**Keywords:** Rat model, Long bone shaft fractures, Mesenchymal stem cells, Hydrogel, Fracture healing

## Abstract

**Background:**

Mesenchymal stem cells (MSCs) are used in cell therapy to enhance healing in long bone fractures, where nonunion is highly likely to occur. However, only a few studies have been conducted on carriers that should be used for more effective cell delivery. This study compared the use of hydrogel and normal saline as carriers for enhancing fracture healing when injecting MSC in a long bone fracture rat model.

**Methods:**

Wistar rats were classified according to the carrier used for MSC injection: Groups C (normal saline) and H (hydrogel). MSCs were administered to the fracture site via direct injection method. The long bone specimens were harvested at 2 and 6 weeks after fracture. Western blot analysis was used to assess the expression of chemokines associated with MSC recruitment (stromal cell-derived factor 1 [SDF-1], monocyte chemoattractant protein-1 [MCP-1]), and osteogenesis (bone morphogenetic protein-2 [BMP-2], transforming growth factor-beta 1 [TGF-β1]) at 2 and 6 weeks post-fracture, respectively. Bone volume [BV], percentage bone volume [PBV] and bone marrow density [BMD]) were evaluated using micro-computed tomography (CT). MSC survival was monitored using fluorescence imaging up to 2 weeks post-fracture.

**Results:**

Group H demonstrated higher SDF-1 and MCP-1 levels at 2 weeks post-fracture compared with group C. No significant differences in protein expression levels of osteogenesis-related chemokines were observed between the two groups at 6 weeks post-fracture. Micro-CT showed higher BV, PBV and BMD in group H compared with group C at 6 weeks post-fracture. Signals of MSCs injected into fracture site lasted longer in group H until 2 weeks post-fracture and showed higher radiance efficiency in each period than in group C.

**Conclusions:**

Compared with using normal saline, using hydrogel significantly enhanced fracture healing in the early phase and formed more new bones at higher densities in the late phase. Injected MSCs were more concentrated around the fracture site and survived longer with hydrogel use than with normal saline use.

**Level of evidence:**

Level V.

**Supplementary Information:**

The online version contains supplementary material available at 10.1186/s13018-025-06029-y.

## Background

Despite continuous advances in treatment methods, painful nonunion occurs in approximately 10% of patients with fractures annually [1]. Especially in long bone fractures, nonunions lead to prolonged treatment periods, which not only increases morbidity [2] but also increases indirect costs, thus causing a socioeconomic burden [3]. Autogenous bone grafting has been used as the gold standard for treating nonunions [4], although it has limitations such as donor site morbidity [5, 6] and difficulty in harvesting sufficient graft material in older patients with osteoporosis [7]. Furthermore, autogenous bone grafting requires an additional surgical procedure, making it difficult to perform concomitantly with the primary surgery [8].

Various cells are involved in the process of fracture healing [9]. In the early phase of fracture healing, cytokines that recruit and proliferate mesenchymal stem cells (MSCs) from immune cells are secreted, preparing the healing phase [10], and fracture healing is then achieved through angiogenesis and osteogenesis processes [11]. In this process, MSCs are a major cellular component for healing that are homed to the fracture area and differentiate into chondrocytes and osteoblasts [12]. The therapeutic potential of MSCs for bone regeneration has been demonstrated [1, 13, 14], and both direct injection into the fracture site and systemic injection through the vein are effective for bone regeneration [15, 16]. However, intravenous injection may have side effects, such as pulmonary embolism, because the injected MSCs are entrapped in the capillaries [17, 18]. Thus, direct injection has emerged as a viable alternative to systemic injection [19].

In previous studies evaluating the therapeutic effects of MSCs by direct injection, normal saline was mostly used as the MSC carrier [20–22]. In an enclosed environment such as the intra-articular space, the use of saline as a carrier does not cause leakage of the injected MSCs [20, 23]. However, in the case of fractures, the fracture site is frequently required to be opened for open reduction and internal fixation and the use of saline as a carrier more likely results in extravasation of the injected MSCs [24]. Therefore, advantages of carriers such as hydrogels, which are more durable at the injury site and can increase the proliferation and retention of MSCs, have been reported [25–27]. However, only a few studies have used them in fracture healing. Furthermore, a study in which exosomes were administered into the fracture area using hydrogel as a carrier only analyzed the impact of exosomes on fracture healing [22], without reporting the results according to the carrier. Therefore, this study aimed to evaluate the effects of direct injection of MSCs using normal saline and hydrogels as carriers on fracture healing in a long bone fracture rat model. We hypothesized that the use of a hydrogel as a carrier enhances fracture healing and retention of injected MSCs at the fracture area compared with the use of normal saline.

## Methods

All procedures involving animals in this study were approved by the Institutional Animal Care and Use Committee of the Clinical Research Institute. Final approval was obtained from the Ethics Committee of Kyung Hee University Hospital at Gangdong (KHNMC AP 2021–010).

### Preparation of bone marrow-derived MSCs (BM-MSCs) and hydrogel

Bone marrow was harvested from 7-week-old male Wistar rats and cultured in 20% Dulbecco’s modified eagle medium (supplemented with 20% fetal bovine serum [FBS], penicillin [100 U/mL], streptomycin [100 µg/mL]; all from Thermo Fisher Scientific). The growth medium was changed every 3 days. Resuspension and recentrifugation were performed when the cells reached 90% confluence, and BM-MSCs at the third passage were used for all experiments. All cultured BM-MSCs were confirmed to be free of mycoplasma contamination using the MycoAlert™ PLUS Mycoplasma Detection Kit (Lonza). A total of 5.0 × 10^6^ BM-MSCs were injected [1], and the cell count was confirmed using a hemocytometer immediately prior to injection.

The HyStem®-HP hydrogel kit (Advanced BioMatrix, Carlsbad, CA, USA), which consists of thiolated-hyaluronic acid, gelatin, and heparin, was used in this study [2]. The breathing method was used to incorporate BM-MSCs after polymerization of the hydrogel [28].

### Categorization according to the carrier and long bone fracture rat model

The rats were randomly categorized into two groups: Groups injected with normal saline (group C, *n* = 6) and hydrogel (group H, *n* = 6). In total, 24 Wistar rats (male, 8 weeks old, weighing 200–250 g,) were subjected to a fracture in the right femoral shaft using a previously described method [1].

All operations were performed by the corresponding author (M.S.K). A total of 5.0 × 10^6^ BM-MSCs were administered directly to the fracture area with 0.1 mL of normal saline and hydrogel, respectively. In group C (with normal saline), the muscular fascia was closed after injection to minimize extravasation. In group H (with hydrogel), the muscular fascia was repaired after injection. The detailed process was conducted in accordance with the methodology described in the previous study [1]. Weight-bearing activities were unrestricted postoperatively. The rats were raised in a controlled environment at 21 ± 2 °C with a 12-h/12-h light/dark cycle.

### In vivo and in vitro studies

The early and late phases of fracture healing were set to 2 and 6 weeks postoperatively, respectively. The rats were sacrificed using a CO_2_ chamber at each time point.

### Radiologic analysis

The volume of newly formed bone was evaluated using micro-computed tomography (micro-CT): bone volume (BV), percentage of bone volume (PBV) and bone mineral density (BMD) [3]. The long bone specimens were scanned three-dimensional (3D) reconstruction of micro-CT images (μCT 80; Scanco Medical, Brüttisellen, Switzerland). A small animal scanning setting (70 kVp, 114 mA, 200-ms integration time) was used for the micro-CT scanning, which resulted in an image pixel size of 19.5 µm. The operator used a two-dimensional preview X-ray (scout view) to determine the region of interest (ROI) to be scanned. This region comprised 254 slices, starting from the first slice containing a 10-mm distance to the proximal bone fracture and moving distally throughout the entire fracture area. The 3D images were smoothed using a 0.5 mm aluminum filter and segmented using specimen-specific global thresholding.

### Histological analysis

Decalcified femur specimens were sagittally sectioned at 5 µm thickness for hematoxylin and eosin (H&E) staining (Sigma-Aldrich). The sections were observed using an microscope (Olympus CX41, Olympus Co., Tokyo, Japan) and the histologic score was calculated [4] (Table 1).

**Table 1:** Histologic scores according to findings of the fracture area

Score	Assessment of histology
1	Fibrous tissue
2	Predominant fibrous tissue with minimal cartilage
3	Equal amount of fibrous and cartilage tissue
4	Predominantly cartilage with minimal fibrous tissue
5	Completely cartilage tissue
6	Predominantly cartilage with minimal immature bone
7	Equal amount of cartilage and immature bone
8	Predominantly immature bone with minimal cartilage
9	Fracture healingwith immature bone
10	Fracture healingwith mature bone

### Western blot analysis

Femur specimens were ground using liquid nitrogen and incubated in lysis buffer containing 140 mM NaCl, 50 mM NaF, 1 mM ethylenediaminetetraacetic acid, 1 mM Na_3_VO_4_, 1 mM phenylmethylsulfonyl fluoride, 10 μg/mL aprotinin, and 1% (w/v) Nonidet P-40 in 20 mM Tris–HCl (pH 7.4). Individual protein were extracted by sodium dodecyl-sulfate polyacrylamide gel electrophoresis and electroblotted to polyvinylidene difluoride membranes (Bio-Rad, Hercules, CA, USA). Subsequently, the membranes were blocked with 5% skim milk in Tris-buffered saline containing 0.1% Tween 20 for 1 h and incubated overnight at 4 °C with primary antibodies and β-actin (Santa Cruz, Cat # sc-47778, 1:5000). The membranes were then incubated for 1 h with anti-goat horseradish peroxidase-conjugated immunoglobulin G (Enzo Life Science, Farmingdale, NY, USA). Immunoreactive bands were detected using the EPD Western Reagent Kit (ELPIS-BIOTECH, Daejeon, Republic of Korea). The protein bands were imaged by a ChemicDoc XRS system and band intensities were measured using the ImageJ software. β-Actin was used as the loading control.

At 2 weeks after fracture, the expression levels of stromal cell-derived factor 1 (SDF-1, Abcam, Cat # ab18919, 1:3000) and monocyte chemoattractant protein-1 (MCP-1, Thermo Fisher Scientific, Cat # PA5-34505, 1:1000), which are related to MSC recruitment, were evaluated. At 6 weeks post-fracture, the expression levels of bone morphogenetic protein-2 (BMP-2, Novus Biologicals, Cat # NBP1-19751, 1:5000) and transforming growth factor-beta 1 (TGF-β1, Abcam, Cat # ab215715, 1:2000), which are related to osteogenesis, were evaluated.

In addition, the vascular endothelial growth factor (VEGF, Santa Cruz, Cat # SC-7269, 1:2000), which is related to vascular regeneration, was evaluated at 2 and 6 weeks post-fracture. Each chemokine was analyzed by evaluating the fold-change in protein expression levels in group H, with group C as the control.

### Distribution and survival for injected BM-MSCs

The distribution and survival of injected MSCs were tracked using the in vivo imaging system (IVIS) spectrum imaging system on the day of injection and 1, 3, 7, and 14 days after injection [5]. BM-MSCs VivoTrack680 labeling was performed according to the manufacturer’s recommendations (PerkinElmer, NEV12000). Cell suspension (10^6^ cells/ml) in phosphate-buffered saline (PBS) was incubated with 50 μg/mL VivoTrack680 for 15 min at room temperature, protected from light, and washed three times with PBS containing 1% FBS. Rats were anesthetized with 1.5% isoflurane (Isoflutek, 710,004) and secured in an IVIS imaging chamber for fluorescence imaging (FLI) of the abdomen or lateral views. For FLI analysis, ROIs were manually designated and analyzed using the Living Image 4.5 software (PerkinElmer). Fluorescence emission data were expressed as radiant efficiency ([p/s/cm^2^/sr]/[μW/cm^2^]), and the color scale was adjusted according to the strength of the signal. Autofluorescence from specific signals was extracted using a spectral unmixing analysis to quantify the fluorescence signal. For semi-quantitative evaluation, an ROI analysis was performed to calculate the accumulation rate of the injected BM-MSCs. The relative signal intensity of the injected BM-MSCs at 1, 3, 7, and 14 days after injection was measured as a percentage of the signal intensity on the day of injection (4–5 h after injection).

### Statistical analysis

The Mann–Whitney test was used to analysis the differences in the volume of newly formed bone, fracture healing scores, protein expression level, and quantified fluorescence signal intensity on IVIS spectrum imaging. Statistical significance was set at *p*-values of 0.05, with a 95% confidence interval. The SPSS version 21.0 software (SPSS, Inc., Chicago, Illinois, USA) was used for all statistical analyses.

## Results

### Fracture healing evaluated by micro-CT analysis

At 2 weeks post-fracture, BV (77.5 ± 22.5 μm^3^ vs. 62.6 ± 12.6 μm^3^, *p* = 0.281), PBV (53.5% ± 5.4% vs. 44.6% ± 11.3%, *p* = 0.203), and BMD (379.0 ± 24.3 mg/cc vs. 338.6 ± 44.8 mg/cc, *p* = 0.152) were higher in group H than in group C, although the difference was not statistically significant.

**Fig. 1:**
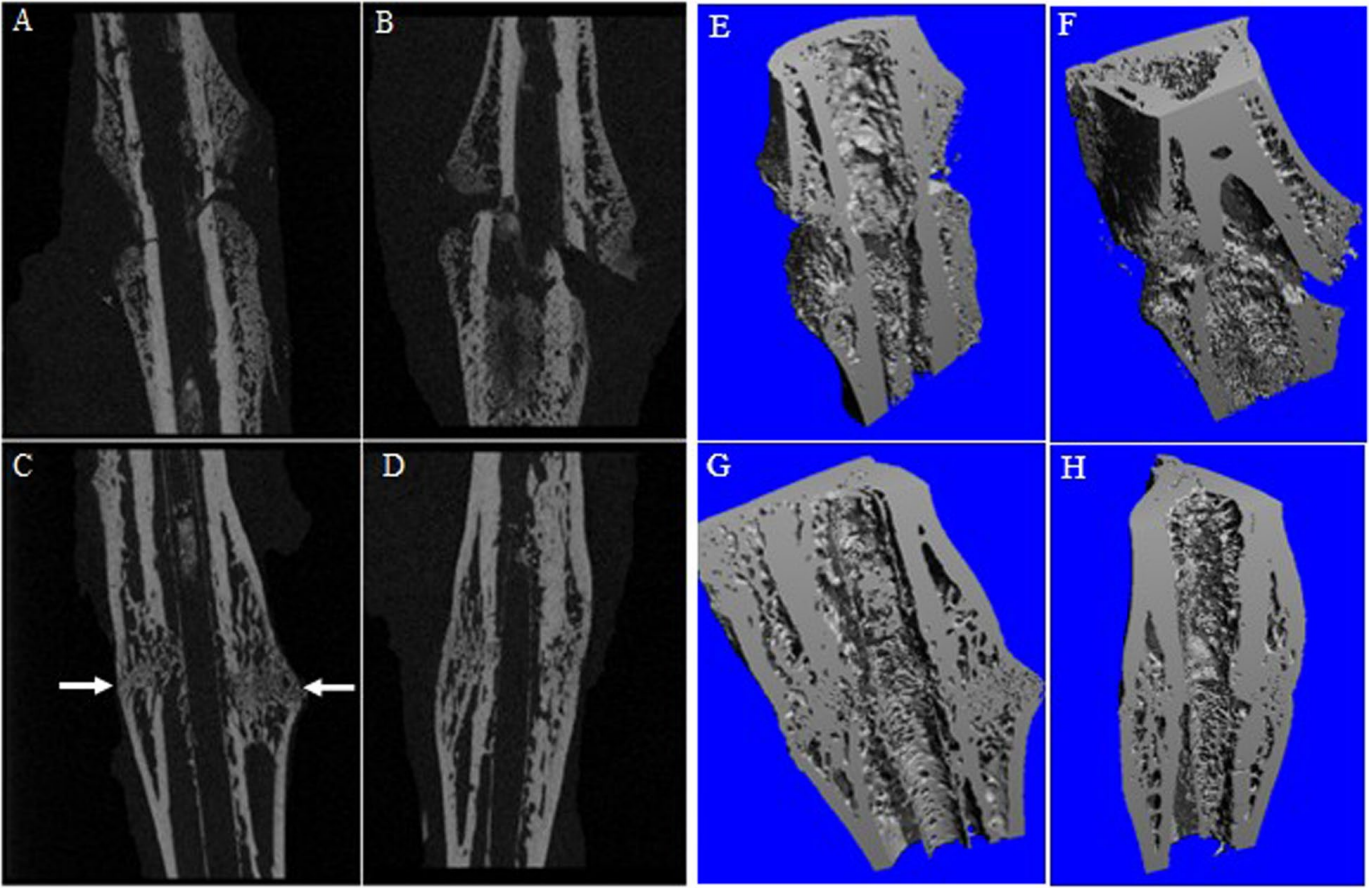
2D and 3D Micro-computed tomography imaging. **A**, **E** Group C, at 2 weeks afterfracture. **B**, **F** Group H, at 2 weeks post-fracture. More newly formed callus can be seen in group H than in group C. **C**, **G** Group C, at 6 weeks post-fracture. **D**, **H** Group H, at 6 weeks afterfracture. In group C, a fracture line is faintly observed (white arrow), indicating that complete union has not yet occurred

At 6 weeks post-fracture, BV (68.0 ± 11.0 μm^3^ vs. 46.9 ± 5.7 μm^3^, *p* = 0.005), PBV (65.0% ± 6.0% vs. 51.4% ± 9.7%, *p* = 0.029), and BMD (693.1 ± 109.5 mg/cc vs. 567.7 ± 41.5 mg/cc, *p* = 0.044) were significantly increased in group H compared with those in group C (Fig. 1).

### Histological fracture healing scores

At 2 weeks after fracture, the fracture healing score was significantly higher in group H than in group C (5.8 ± 1.6 vs. 3.4 ± 1.5, *p* = 0.043), and more fibrous and cartilaginous tissues were observed in group H under the microscope.

**Fig. 2:**
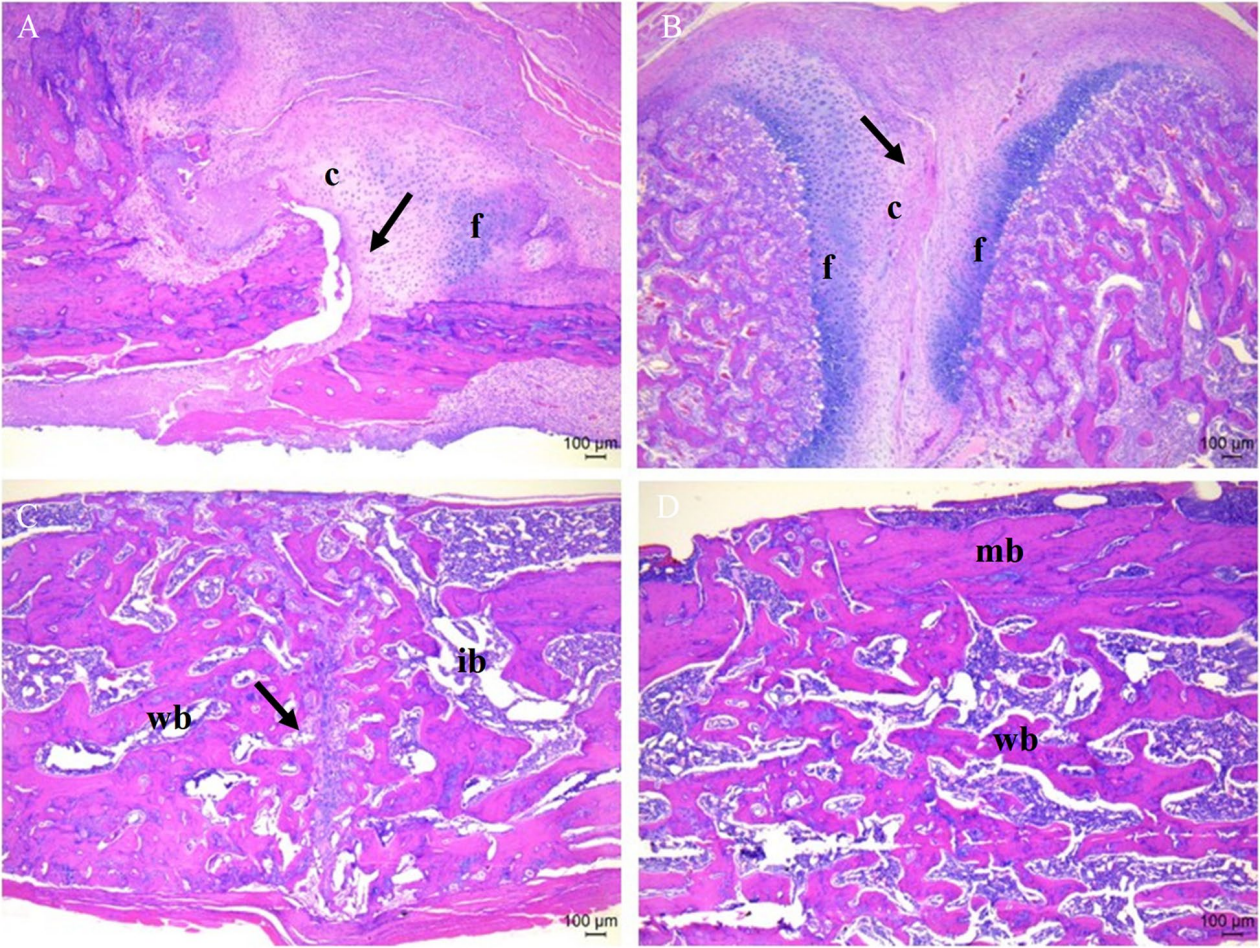
Histologic analysis for fracture healing (40 × magnification). **A** Group C, at 2 weeks post-fracture. **B** Group H, at 2 weeks post-fracture. **C** Group C, at 6 weeks post-fracture. **D** Group H, at 6 weeks post-fracture. The black arrows points to the fracture lines. Scale bar = 100 μm. c, cartilage in the fracture area; f, fibrous tissue; wb, woven bone; ib, immature bone; mb, mature bone

At 6 weeks after fracture, no significant difference in fracture healing scores was observed between groups H and C (9.8 ± 0.4 vs. 9.6 ± 0.5, *p* = 0.545). Immature and mature bones were observed in both groups under a microscope, although some fracture lines were observed in group C (Fig. 2).

### Expression levels of chemokines associated with MSC recruitment, osteogenesis, and angiogenesis depending on the phase of fracture healing

**Fig. 3:**
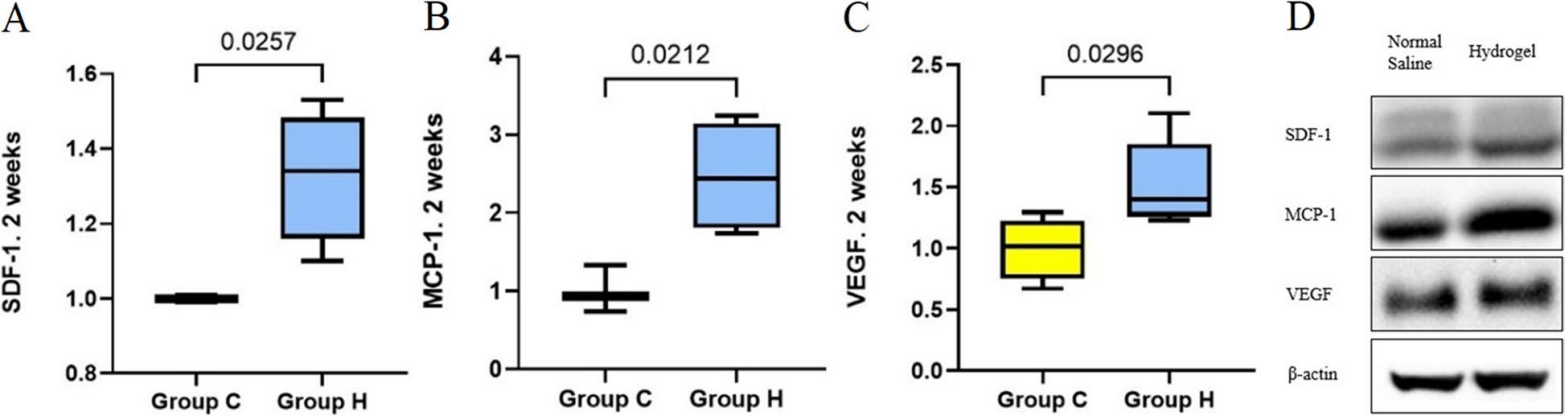
Expression levels of chemokines related to mesenchymal stem cell recruitment and angiogenesis at 2 weeks after fracture. **A** SDF, stromal cell-derived factor **B** MCP-1, monocyte chemoattractant protein-1 **C** VEGF, vascular endothelial growth factor **D** Western blot band

At 2 weeks after fracture, the fold changes in the mRNA protein expression levels of SDF-1 and MCP-1 were significantly higher in group H than in group C (*p* = 0.026 and *p* = 0.021, respectively). VEGF expression levels were significantly higher in group H than in group C at 2 weeks after fracture (*p* = 0.030) (Fig. 3).

**Fig. 4:**
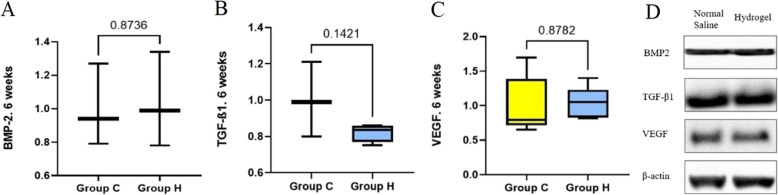
Expression levels of osteogenesis-related factors and chemokines related to angiogenesis at 6 weeks after fracture. **A** BMP-2, bone morphogenetic protein 2 **B** TGF-β1, transforming growth factor-beta 1 **C** VEGF, vascular endothelial growth factor **D** Western blot band

At 6 weeks after fracture, no significant differences in BMP-2, TGF-β1 and VEGF expression levels were observed between groups H and C (*p* = 0.874, *p* = 0.142 and *p* = 0.878, respectively) (Fig. 4) (Supplementary Data 1).

### FLI at day of injection and 1, 3, 7, and 14 days after injection

**Fig. 5:**
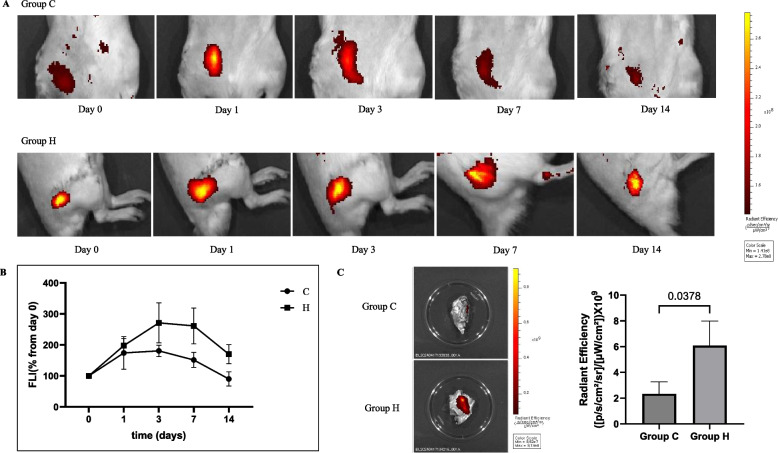
Distribution and survival of administered bone marrow-derived mesenchymal stem cells. **A** In vivo imaging system spectrum imaging. **B** Flow of fluorescence signal intensity over time after injection. **C** Ex vivo fluorescence imaging 14 days after injection

At 1, 3, 7, and 14 days after injection, the signal intensity of the injected BM-MSCs was higher in group H than in group C. Furthermore, at 14 days after injection, the signal intensity of the injected BM-MSCs was significantly higher in group H compared to group C in the ex vivo FLI of harvested specimens (*p* = 0.038) (Fig. 5).

## Discussion

In this study, fracture healing was more enhanced in the group injected with MSCs using hydrogel as a carrier (group H) than in the group injected with normal saline as a carrier (group C). In the in vivo analysis, group H demonstrated more bone formation both 2 and 6 weeks after fracture and significantly higher fracture healing scores at 2 weeks post-fracture than group C. The SDF-1 and MCP-1 expression levels, associated with the homing of MSCs, were significantly higher in group H than in group C at 2 weeks after fracture in vitro. In addition, in vivo and ex vivo IVIS spectrum imaging revealed higher signal intensity of MSCs injected into the fracture area and longer retention of MSCs in group H than in group C.

MSCs are major cells that affect bone healing [6, 7], and studies have reported that fracture union or bone formation is accelerated after MSC injection in several animal models [8, 9]. However, few studies have analyzed the optimal methods for injecting MSCs to enhance fracture healing before applying them in the clinical field. Prior to this study, an animal study has reported on the optimal concentration of MSCs required to promote bone healing [1]; however, no studies have identified which method of injection is more effective.

Two methods for injecting MSCs have been introduced: localized injection at the fracture site and systemic intravenous injection [12]. Systemic intravenous injection of MSCs is primarily used in animal studies because it is less invasive [3, 9, 13]. However, systemic injection of MSCs can lead to severe complications, such as pulmonary embolism [14] and injected cells may be trapped in the lungs, inhibiting MSC migration to the injured site [15]. Hence, in an animal study, Mohammed et al. have reported that the direct injection of MSCs accelerated bone healing more than the systemic injection of MSCs [12]. Moreover, in the clinical field, the majority of patients with long bone fractures undergo surgical fixation of the fracture [16], and MSCs can be directly injected into the fracture site concurrently with access to the fracture site [17]. Therefore, animal studies on direct injection are required for the clinical application of MSCs to accelerate fracture healing.

In previous studies, the carriers used for direct injection of MSCs were mostly normal saline [18–20], with other materials such as hydrogels and scaffolds occasionally used [21, 22]. The homing effect, in which MSCs are homed to the fracture area and undergo differentiation, is a critical factor in accelerating bone healing [7]. The more exogenous MSCs recruited to the fracture area, the more bone healing was promoted [23]. Therefore, it is necessary to determine the carrier that is most effective at prolonging the retention of injected MSCs at the fracture site when direct injection is performed. Normal saline, which has been used primarily as a carrier, may reduce the number of cells delivered to the fracture site owing to leakage [24], whereas hydrogels are suitable for treating bone-related diseases because of their stability, prolonged retention at the target site, and capacity to deliver encapsulated MSCs [25]. However, no study has compared the two carriers. In this study, factors related to the recruitment of MSCs in the early phase were evaluated after MSCs were injected with normal saline and hydrogel as carriers in a long bone fracture rat model. The SDF-1 and MCP-1 expression levels were significantly higher in the group using hydrogel as a carrier than in the control group. Furthermore, in the late phase, BMD was significantly higher in the group that used the hydrogel as a carrier than in the control group. Thus, the ratio of newly regenerated bone callus to total callus was greater in the hydrogel group than in the normal saline group.

The localization and persistence of the administered cells are critical factors affecting bone healing [26]. Consequently, FLI has been used in previous studies as a tool for tracking the distribution and survival of transplanted MSCs [3, 27]. Although not in a fracture model, Preda et al. analyzed the lifespan of transplanted MSCs using the IVIS spectrum imaging system and reported that when injected directly into the target organ, a signal persisted in the target organ at similar intensities for 7 days after injection, which subsequently decreased. They also reported that when the MSCs were injected intravenously, they were observed in tissues other than the target organ, and the signal decreased after 3 days [27]. In this study, in vivo and ex vivo IVIS spectrum imaging showed a decrease in the signal 3 days after injection in group C. In group H, the signal intensity decreased after 7 days of injection, indicating that MSCs were more distributed to the fracture site and survived longer with hydrogel use. Hoffman et al. have reported that transplanting MSCs into an allogeneic bone graft using a hydrogel resulted in enhanced localization of cells and longer survival compared with direct seeding [26], demonstrating results similar to those observed in this study.

This study had several limitations. First, only normal saline and hydrogels were evaluated as carriers for MSC injection. Hydrogels are useful for applications in bone-related diseases because they have good biocompatibility and biodegradability. However, in nonunion models with segmental bone defects, a construct with a volume such as a scaffold may be required. Second, although BMP levels were quantitatively evaluated using western blot analysis, the localization and distribution of BMP were not evaluated using analyses such as immunohistochemistry. In addition, osteoclast activity was not evaluated through TRAP staining. Third, although the distribution and survival of the injected MSCs were assessed using the IVIS spectrum imaging system, a more objective quantitative analysis was not performed at each time point. Further evaluations such as single cell analysis of newly formed callus should be performed in the future. Therefore, MSC-derived apoptotic bodies may be engulfed by macrophages, resulting in false-positive results. Nevertheless, this study demonstrates that the use of hydrogel as a carrier for the injection of MSCs was more effective for enhancing healing of long bone fractures than the use of normal saline through in vivo and in vitro analyses and FLI. The findings will contribute to the future clinical use of direct injection of MSCs.

## Conclusion

When MSCs were directly injected using the hydrogel, fracture healing was significantly enhanced compared with using normal saline in the early phase. In the late phase, when MSCs were injected using a hydrogel as a carrier, significantly more new bone was formed at a higher density than that with normal saline. When MSCs were injected using a hydrogel as a carrier, the injected MSCs were more concentrated around the fracture site and survived longer than those injected using normal saline.

## Supplementary Information

Supplementary Material 1.

## Data Availability

Data is provided within the manuscript or supplementary information files.

## Abbreviations

MSCs: Mesenchymal stem cells
SDF-1: Stromal cell-derived factor-1
MCP-1: Monocyte chemoattractant protein-1
VEGF: Vascular endothelial growth factor
BMP-2: Bone morphogenetic protein-2
TGF-β1: Transforming growth factor-beta-1
BV: Bone volume
PBV: Percentage bone volume
BMD: Bone marrow density
BM-MSCs: Bone marrow-derived MSCs
FBS: Fetal bovine serum
Micro-CT: Micro-computed tomography
3D: Three-dimensional
ROI: Region of interest
H&E: Hematoxylin and eosin
IVIS: In vivo imaging system
PBS: Phosphate-buffered saline
FLI: Fluorescence imaging
