# Supplementary material for: Comparison of the effects of hydrogel and normal saline as carriers of MSC on fracture healing in a rat long bone fracture model
Source: J Orthop Surg Res. 2025 Jul 8;20:629. doi: 10.1186/s13018-025-06029-y (PMC12239429; doi:10.1186/s13018-025-06029-y)
Supplement: Supplementary file 1 — Supplementary Material 1. [file 13018_2025_6029_MOESM1_ESM.docx]

**Supplementary Data 1. Comparison of the protein expression levels of SDF-1, MCP-1, BMP-2, TGF-β1, and VEGF**

| 2 weeks post-fracture | Group C | Group H |
| --- | --- | --- |
| SDF-1 (fold change) | 1 | 1.30±0.18* |
| MCP-1 (fold change) | 1 | 2.46±0.70^*^ |
| VEGF (fold change) | 1 | 1.53±0.34* |
| 6 weeks post-fracture | Group C | Group H |
| BMP-2 (fold change) | 1 | 1.04±0.29 |
| TGF-β1 (fold change) | 1 | 0.82±0.05 |
| VEGF (fold change) | 1 | 1.04±0.23 |

SDF, stromal cell-derived factor; MCP-1, monocyte chemoattractant protein-1; VEGF, vascular endothelial growth factor; BMP-2, bone morphogenetic protein 2; TGF-β1, transforming growth factor-beta 1.

*p < 0.05.
